# To Grasp the World at a Glance: The Role of Attention in Visual and Semantic Associative Processing

**DOI:** 10.3390/jimaging7090191

**Published:** 2021-09-20

**Authors:** Nurit Gronau

**Affiliations:** Department of Psychology and Department of Cognitive Science Studies, The Open University of Israel, Raanana 4353701, Israel; nuritgro@openu.ac.il

**Keywords:** visual attention, spatial attention, scene perception, object recognition, semantic associations, feature-based attention

## Abstract

Associative relations among words, concepts and percepts are the core building blocks of high-level cognition. When viewing the world ‘at a glance’, the associative relations between objects in a scene, or between an object and its visual background, are extracted rapidly. The extent to which such relational processing requires attentional capacity, however, has been heavily disputed over the years. In the present manuscript, I review studies investigating scene–object and object–object associative processing. I then present a series of studies in which I assessed the necessity of spatial attention to various types of visual–semantic relations within a scene. Importantly, in all studies, the spatial and temporal aspects of visual attention were tightly controlled in an attempt to minimize unintentional attention shifts from ‘attended’ to ‘unattended’ regions. Pairs of stimuli—either objects, scenes or a scene and an object—were briefly presented on each trial, while participants were asked to detect a pre-defined target category (e.g., an animal, a nonsense shape). Response times (RTs) to the target detection task were registered when visual attention spanned both stimuli in a pair vs. when attention was focused on only one of two stimuli. Among non-prioritized stimuli that were not defined as to-be-detected targets, findings consistently demonstrated rapid associative processing when stimuli were fully attended, i.e., shorter RTs to associated than unassociated pairs. Focusing attention on a single stimulus only, however, largely impaired this relational processing. Notably, prioritized targets continued to affect performance even when positioned at an unattended location, and their associative relations with the attended items were well processed and analyzed. Our findings portray an important dissociation between unattended task-irrelevant and task-relevant items: while the former require spatial attentional resources in order to be linked to stimuli positioned inside the attentional focus, the latter may influence high-level recognition and associative processes via feature-based attentional mechanisms that are largely independent of spatial attention.

## 1. Introduction

Associative relations among concepts or percepts have been at the heart of psychological investigation since early research days. Within the visual domain, the associative relations between global scene structures and individual objects embedded in them, or between objects themselves, have received great interest in recent years. How fast do we grasp the ‘gist’ or the core meaning of a scene? How many objects within a scene can we simultaneously recognize? Can we immediately perceive the relations among objects and can we spot any oddities, such as an object that does not ‘fit’ within its contextual surrounding?

When viewing a complex scene during a brief glimpse, our cognitive system faces serious capacity limitations in an attempt to process such rich information. One factor that may reduce scene complexity and streamline visual recognition is attention, a selection mechanism that allows people to focus on high-priority stimuli and to filter out or attenuate the processing of material of secondary importance (e.g., [1,2,3,4]). Attention leads our experience to be dominated by one thing rather than another, such as a salient stimulus that ‘jumps to the eye’ or an object that we chose to focus on at a certain moment. Specifically, within the visual modality, *spatial attention* is thought to shift from one region in the visual field to another, by explicitly moving the eyes (e.g., in order to focus our gaze on a specific item), or by covertly orienting processing resources to a region/object (or even a feature), regardless of eye position [5]. When discussing scene understanding under very brief viewing conditions, however, such shifts of attention and the eyes from one region to another are typically prevented. Consequently, the ability of our cognitive system to compute semantic relationships within the scene, when individual objects, or whole scene parts, appear *outside* one’s attentional ‘spotlight’, is questionable. Can we recover the content of these unattended regions and stimuli, and can we successfully process their associative relations (e.g., with items positioned ‘inside’ the main focus of attention), despite their underprivileged status?

Traditional attention models have posited that at any moment only one or perhaps a very small number of objects can be attended and processed to a level of full recognition (e.g., [6,7,8]). Supposedly, the attended stimuli can be linked together, such that their visual and semantic relations are instantly grasped (e.g., ‘there is a computer screen, standing on a table, within a larger office scenario’). However, what is the fate of stimuli that do not fall within the focus of attention (e.g., a printer, located at the other side of the room)? Objects appearing outside the main focus of attention, according to conventional models, are largely filtered out, and/or are processed only to a coarse level (e.g., [9]). Salient changes to unattended objects may be overlooked and the construction of a global scene representation may be incomplete (e.g., [10,11,12]). Accordingly, the perception of the relations between unattended stimuli, or between attended and unattended objects, is very minimal.

In contrast, accumulating evidence in the last two decades has offered a different perspective on unattended visual processing. Recent studies have suggested that during a brief visual glance, real-world objects that are learnt and familiarized through lifetime experience (as opposed to meaningless or arbitrary stimuli used in many of the traditional studies) form a unique class of stimuli that can be detected and recognized even in the near absence of visual attention. In a series of studies using a dual task paradigm, participants performed a demanding central task while at the same time successfully detected a predefined object category (e.g., an animal or a vehicle) appearing at an unattended (i.e., peripheral) location [13,14]. Follow-up studies have revealed that tasks requiring even much finer object discriminations (e.g., ‘Is a peripheral object a car or a non-car vehicle?’, or ‘Is a peripheral face of Tom Cruise or not?’) can be additionally performed under such minimally attended conditions ([15,16,17] see also [18]). Based on these findings, the authors concluded that high-level object representations are formed in parallel and can be accessed even when stimuli appear outside the main focus of spatial attention. Accordingly, one may assume that real-world objects are recognized and linked with each other in the absence of focal attention (but see opposing findings in [9,19,20,21,22].

Note that the aforementioned studies have mainly focused on the identification of individual, isolated items, positioned in peripheral (unattended) visual locations. In doing so, these studies have provided important input concerning the necessity of attention in the recognition of some of the very building blocks of scenes—individual objects or even specific stimulus features. A somewhat different perspective to the question of attentional requirement to visual and semantic associative processes, however, arises from studies directly examining relational processing during scene viewing. In the present paper, I will review such research investigating the processing of associative relations between large-scale scenes and the objects embedded within them, as well as between individual objects themselves. Then, I will present my own perspective to the question of the necessity of spatial attention to visual-semantic associative processes, based on accumulating research in my lab.

## 2. Processing Scene–Object Associative Relations

Scenes are rich visual structures that can be characterized by large-scale properties, such as spatial layout, surface and color, as well as the local objects embedded within these structures. Ample research investigating the perception of natural scenes has suggested that when using very brief exposure durations (e.g., ranging 20–100 ms), subjects can report relatively little detail, yet they are able to grasp the main theme or the ‘gist’ of the scene [23,24,25,26,27,28,29]. For instance, when viewing a scene image for an extremely short duration, participants extract coarse sensory information, such as the general shape or the amount of light and darkness in the image. This input can contribute to successful superordinate categorization of the image (e.g., ‘an outdoor scene’). With somewhat longer exposures, one may grasp additional global features, allowing a finer, basic-level categorization (e.g., ‘a forest’, see [25,26,30]). Still, many important visual details are overlooked and the extraction of specific object identities is often compromised (for recent findings, see [31]).

Nevertheless, several studies have suggested that the rapid extraction of a scene’s gist provides enough information to create a preliminary semantic template of the world, which can guide attention to areas of interest within the scene (e.g., [32,33]). Furthermore, grasping a scene’s main theme can facilitate object recognition by pre-activating or priming associated objects embedded within the scene to a degree that is sufficient for their partial recognition (e.g., [34,35,36,37,38,39,40,41,42]; but see different views on this issue in [43,44,45]). Support for this approach comes mainly from studies examining the effects of scene recognition (e.g., a farm) on the processing of contextually consistent (e.g., a cow) or inconsistent objects (e.g., a vacuum cleaner) embedded in the scene. Such studies have found that participants better recognize objects that conform to their overall context than ones that disagree with it. This contextual integration, or the ability to perceive a coherent visual setting despite brief viewing conditions, may take place even if a contextually consistent object appears in a peripheral visual location, i.e., outside the main focus of visual attention. Objects that conform to a scene’s context, thus, may benefit from reduced recognition thresholds due to top-down processing. Such processing relies on prior expectations and knowledge regarding the objects’ category, their general content or their location in space (see, e.g., [35,46]; but see [47,48] for a critical view on top-down effects on perception). According to some researchers, scenes and objects are processed in parallel, hence not only a scene can rapidly activate an object, but an object can also activate a background scene. Scene–object facilitative effects, therefore, may be bidirectional (see, e.g., [25,42,49,50,51]), and may contribute to an integrated percept of the visual world, even among objects that are positioned at minimally attended locations. Spatial attention, according to this view, may not be essential for visual associative processes during a brief glance (see also [52]).

Paradoxically, however, an opposing approach states that rather than contextually consistent objects being facilitated by prior knowledge and expectation, contextually unassociated or *inconsistent* stimuli are in fact prioritized by the cognitive system when presented outside the main focus of spatial attention. Clearly, such objects are of great informative value since they may violate expectations and possess important novel information that requires elaborated processing and immediate action. While the precise mechanism underlying their rapid prioritization is yet unclear (assuming that they do not differ in their visual saliency and/or perceptual conspicuousness from contextually consistent stimuli), several researchers have proposed that such semantic oddities can be computed pre-attentively. Namely, an object that is semantically inconsistent with its surrounding environment (e.g., a vacuum cleaner in a farm) may rapidly ‘pop-out’ and capture attention (e.g., [51,53,54]). Using a spatial probe method, for instance, Gordon [55] demonstrated that within a short visual glance, attention is preferentially allocated to contextually inconsistent objects rather than to consistent objects within a scene. Furthermore, studies using the change detection flicker paradigm [10] have repeatedly shown an earlier detection of a rapid change to inconsistent than to consistent objects [56,57,58,59]. Interestingly, however, opposite findings (in which a consistency advantage was observed) have been obtained when object identification rather than mere detection was assessed [58,59,60]. Yet, studies using eye movement measures have further provided evidence that scene–object inconsistencies may attract eye fixations when a contextually inconsistent object appears at an extrafoveal location [51,53,54,57,61,62,63,64]. Given the tight correlation between foveal processing and visual attention in everyday vision (see, e.g., [65,66,67]), these findings suggest that semantic oddities can be rapidly computed when appearing within unattended or minimally attended locations. More generally, scene–object associative relations can be at least partially recovered when stimuli are presented outside the main focus of spatial attention.

Note, however, that other eye movement studies have yielded contradictory results, demonstrating no evidence for rapid extrafoveal detection of contextually inconsistent objects within scenes [68,69,70,71,72,73]. According to these latter studies, scene–object inconsistency effects may stem from processes occurring subsequent to, rather than prior to, the allocation of focal attention and the eyes to an object in the scene. Namely, if contextual inconsistencies are incidentally detected during serial scanning of a scene, they may elicit longer dwelling times in an attempt to recover their odd nature (the *attention disengagement hypothesis*, [45,56]). They do not, however, cause an immediate, involuntary shift of spatial attention to their location due to their ‘conceptual saliency’ or their semantic significance (see also [58,74]). According to this view, scene–object inconsistencies are not detected in the absence of spatial attention, but only once attention and the eyes have incidentally ‘landed’ on them, allowing sufficient processing resources for their recovery.

Notably, while the dispute concerning the status of consistent/inconsistent stimuli has a rich history in the scene-perception literature, as well as the attentional literature, a related yet somewhat independent debate has emerged in recent years among researchers studying the processes taking place under unconscious or visually unaware conditions. Admittedly, according to at least one dominant view, attention is necessary for conscious perception and the two processes are tightly interlinked (e.g., [11,75,76], but see [77,78]). It is not surprising, thus, that questions of similar nature have arisen among researchers studying these allegedly separate fields—attention and consciousness. As with research within the attention domain, one class of findings has provided support for the unconscious processing of semantically related objects, namely, objects that conform to prior knowledge and are generally consistent with their contextual environment (see, e.g., [79]). According to an opposing dominant view, however, it is contextually inconsistent stimuli that actually benefit from increased access to awareness and therefore may exert influence on performance earlier than consistent stimuli when presented unconsciously [80] (see also [81,82]). These findings, though, were recently questioned by studies failing to replicate them, forming a relative consensus according to which there is essentially no or very limited integrative (associative) processing under unconscious conditions [83,84,85,86].

It appears, then, that the question of the necessity of attention to scene–object associative processing is tightly linked with, and may have significant implications for, processes beyond mere perception and visual recognition. As mentioned above, one class of studies has provided support for a contextual consistency advantage during unattended processing, whereas another demonstrated an opposite, inconsistency advantage. There is also, however, a third approach to the question of pre-attentive integrative processing. According to such approach, *neither* type of contextual consistencies is in fact prioritized by the cognitive system, since scene–object associations are simply not accessed (or are, at most, minimally accessed) in the absence of attention. As with the visual awareness literature, this view is supported by a bulk of null findings accumulating across studies and methodologies. I will return to this approach, and more generally to studies demonstrating a reliance of visual associative processing on spatial attention, when reviewing my own research.

## 3. Processing Associative Relations among Individual Objects

While scenes form a special case in which objects may be pre-activated due to rapid global processing of a scene’s gist (e.g., [28,30,87]), a different approach to the study of associative processing during scene viewing is to examine the influence of an individual object (rather than a whole scene) on the recognition of a nearby object. Indeed, when stripping off background information and focusing on local object-to-object relations, semantic, categorical and visual associations among individual items are efficiently extracted when stimuli are only glanced for a very brief duration (e.g., [36,88,89,90,91,92,93,94]). The spatial relations between the objects, i.e., whether they are positioned in plausible or implausible locations with respect to each other, further affect the accuracy and speed of recognition performance (e.g., [91,95,96], see also [97,98,99] for related fMRI findings).

Yet, what is the role of spatial attention in analyzing and understanding these relations? In most of the aforementioned studies, stimuli were presented within the main focus of spatial attention, or to the least, there was no attempt to control for attentional factors. Therefore, one could not determine whether significant associative processing actually occurs in the absence of attention. One exception is a recent study, in which semantically associated object images served as irrelevant backgrounds to target letters. Findings showed that participants dwelled longer on pairs of related than on unrelated images. This semantic bias, however, was contingent on the objects being presented for long exposure durations and on attention being explicitly directed to them [100]. These findings are in accordance with classical attention models arguing for the necessity of visual attention to the identification of complex objects and their relations (e.g., [6]).

## 4. A Possible Account for Prior Contradictive Findings: Lack of Control over Spatial Attention?

The findings reviewed above portray a rather confusing picture with respect to the role of spatial attention in visual associative processing. Studies examining object-to-object relational processing have typically presented stimuli to the focus of visual attention, thus they cannot teach us much about one’s ability to grasp object relations with minimal attentional capacity. Studies using scene–object consistency paradigms have often presented stimuli in peripheral (unattended) locations, yet the findings obtained from these studies were highly contradictive. It appears that part of the difficulty in interpreting these mixed results is the lack of a stringent control over spatial attention in many of the experimental paradigms described. As implied earlier, such a lack of control may cause a confusion between pre- and post-attentional processes, e.g., early spatial attentional capture vs. later attentional engagement. Specifically, scene–object consistency paradigms using rather long viewing exposures (including many change detection paradigms characterized by repeated presentation of the same scene image) are particularly prone to this ‘early vs. late’ confusion, due to attention (and the eyes) freely scanning the visual display and potentially latching onto objects of interest once these have been detected. Indeed, when presenting images for very brief exposure durations, the use of a distributed attentional mode allows the extraction of the main theme of a scene (e.g., [23,27,29,101]). However, the detection and identification of a stimulus that violates the overall context (and perhaps even one that conforms to it) likely take place with additional exposure time required for scene scanning (e.g., [46,58]). Even when focusing attention on a specific item, a prolonged scene exposure may allow a ‘slippage’ (i.e., an inadvertent shift, see, e.g., [102,103]) of spatial attention and of eye fixations to more peripheral scene areas.

To prevent this attentional ‘slippage’, one may need to assess visual associative processes under tighter attentional conditions, in which visual attention is controlled both spatially (i.e., by focusing the ‘spotlight’ of attention in advance on a region or an object) and temporally (i.e., by using limited perceptual exposure). Under such conditions, any interference (or facilitative effect) created by a stimulus positioned outside the main focus of attention can imply processing of its relations with an attended item under truly ‘unattended’ conditions. Surprisingly though, very few studies have utilized focused attention paradigms, in which scene/object exposure durations have been tightly controlled.

In a series of studies conducted in my lab, we adopted such an approach to investigate the necessity of spatial attention to relational processing during a brief visual glance. Specifically, we used pairs of stimuli (e.g., two objects, two scenes, or an object and a scene) to examine the rapid perception of their relations (e.g., consistent/inconsistent, categorically related/unrelated). The assessment of these associative relations was performed under conditions in which stimuli were both attended, compared to conditions in which one of two stimuli was unattended, or minimally attended. Note that the use of the terms ‘attended’ and ‘unattended’ may be somewhat misleading, as it may imply that there is a clear dichotomy between the two types of states. Spatial attention, however, is typically portrayed as a distribution of activation peaking around the focus of interest and gradually decreasing with distance (e.g., [104]; for a more elaborated model, see, e.g., [105]). For illustrative purposes, I will nevertheless use these allegedly dichotomous terms to represent perceptual processing within the *main focus* of spatial attention vs. processing within its *outskirts*. We assumed that when both stimuli were spatially attended, participants could easily grasp the relations between them, yielding faster and more accurate responses to contextually consistent than to inconsistent pairs. Our main question of interest, however, concerned a situation in which spatial attention was focused on only one of two stimuli, while its counterpart item was presented ‘outside’ the attentional focus. Would we still observe a contextual consistency effect, implying associative processing even when one of the stimuli was unattended? To foreshadow our results, no such processing was observed in the absence of spatial attention, suggesting that the latter is necessary for visual–semantic associative processes. Importantly, however, if an item (e.g., object) was actively searched for by the observers (e.g., when defined as a task-relevant target), it affected performance and was associatively linked to an attended object, regardless of its spatial location (inside/outside the attentional focus). Presumably, the prioritization of specific visual features via task demands allowed a to-be-detected target to ‘break’ through the spatial attention gating mechanism and access high-level processes, even when positioned in an unattended location. This apparent orthogonality of *spatial-based* and *feature-based* attentional mechanisms will be further discussed below.

In the next section, I will describe our experimental paradigm with more detail to allow a better understanding of the research rationale and our main findings.

## 5. The Necessity of Spatial Attention to Processing Contextual Relations among Everyday Objects

**Figure 1:**
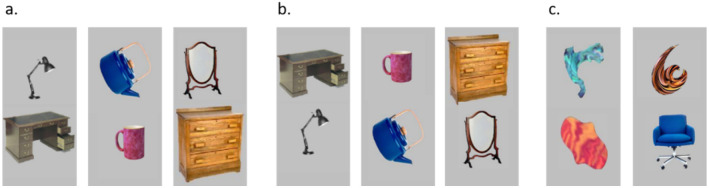
Examples of (**a**) spatially consistent and (**b**) spatially inconsistent pair objects. (**c**) Nonsense target shapes, paired with each other or with a real-world object.

**Figure 2:**
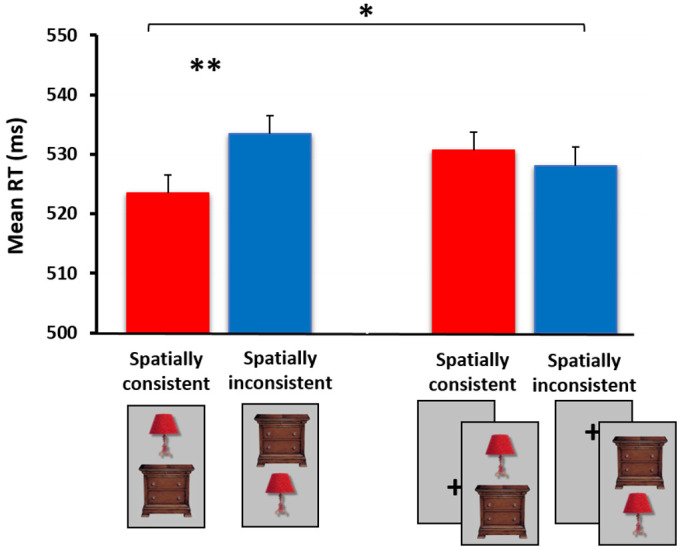
The spatial consistency effect among the nontarget stimuli in the basic experimental version (i.e., both stimuli are attended, left) vs. the spatial cuing experimental version (i.e., only one of two stimuli is fully attended and relevant to task requirements, right). The cross seen on the right served as a spatial cue, flashing immediately before stimuli’s appearance, either in an upper (50%) or a lower location. In order to validate the spatial cuing manipulation, an additional condition (not shown here) was added to the experiment, including a single object—either cued or uncued—on which the stimulus classification task was performed. Obtaining faster RTs to the cued items assured that participants indeed responded to the spatial cue and shifted their visual attention to the cued location. Standard errors presented on top of the bars were computed for the difference RT scores (i.e., spatially inconsistent—consistent, see, e.g., [106]) within each experiment. A statistically significant between-subject interaction was obtained when comparing the results of the two experiments (N = 20 in each). * *p* < 0.05, ** *p* < 0.01. The full results are published in [96].

Objects tend to appear within specific environments (e.g., a desk and a computer screen, within an office) and in specific relative spatial positions (e.g., the screen sitting *above*, not below, the desk). In our first set of experiments [96], we examined attentional involvement in contextual integration processes, and specifically in the perception of ‘correct’ spatial relations among everyday objects. Participants were presented with pairs of semantically associated objects that either appeared in their correct/expected relative spatial positions (e.g., a lamp on top of a night stand) or in incorrect/unexpected positions (e.g., a lamp under a night stand). On part of the trials, the spatially consistent/inconsistent pairs did not appear, and instead, a nonsense shape was presented, paired with another nonsense shape or with a real-world object (see Figure 1). The participants’ task was to determine as fast as possible whether each trial contained a nonsense target stimulus, or not, by using a forced-choice response. Importantly, stimuli in each trial were presented for a very brief duration (approximately 60 ms.) and were masked by a pseudo-noise pattern image. Since the nonsense target shape could potentially appear in each of the two stimulus locations (i.e., upper or lower), participants presumably oriented their ‘spotlight of attention’ to both stimulus locations in order to detect the nonsense target. We therefore considered this situation as an *attended* one, in which both stimuli fell ‘inside’ the main focus of attention. Note that instruction-wise, participants were tuned to detect the nonsense shape, but in fact responses to this type of target were only of minor interest. Our main interest was in participants’ responses to the nontarget trials containing the (non-prioritized) pairs of objects positioned in spatially consistent or inconsistent relative locations. Participants were not informed in any way that we were interested in their responses to these stimuli and/or to contextual integration processes. It was thus the *incidental* encoding of object-to-object associative relations that was the focus of the research (I will return to this point later on). As mentioned earlier, we hypothesized that despite their irrelevance to task demands, these spatial relations would be readily processed and would elicit shorter reaction times (RTs) among spatially consistent than inconsistent object pairs. Namely, pairs of objects that conform to one’s knowledge of the world (or one’s *schema*) would be processed more efficiently than pairs that disagree with it. Indeed, the results showed that this was the case, suggesting that participants were sensitive to the objects’ associative relations when both stimuli were attended (see Figure 2, left).

Next, we ran an experiment in which we manipulated participants’ allocation of spatial attention in order to determine whether the processing of the associative relations among the objects necessitated attention. Using a similar visual display to that of the previous experiment, we now added a spatial cuing manipulation that summoned participants’ attention, prior to stimuli appearance, to *one* of the two object stimuli in each pair. This was performed by flashing a brief cross in one of the two locations, immediately before stimuli’s appearance. Task instructions were additionally changed such that the object classification task was now performed on the cued stimulus only, instead of on both stimuli within a pair (e.g., ‘if there is a nonsense shape in the *cued location* press “1”, otherwise press “2”‘). As a result, the cued object was fully attended and task-relevant, while the uncued object (now falling ‘outside’ the main focus of spatial attention) became a task-irrelevant distractor that could be effectively ignored. We asked whether a spatial consistency effect would be obtained with the real-world (nontarget) objects under such conditions, in which one of the two stimuli was unattended, or at most, minimally attended. To the extent that a spatial consistency effect was observed, we could conclude that object-to-object contextual relations were processed despite the underprivileged attentional conditions of one of the two stimuli. Obtaining no spatial consistency effect, in contrast, would strongly suggest that linking the two objects within a more global contextually coherent percept necessitates attentional capacity.

Interestingly, the spatial consistency effect observed in the previous experiment was completely eliminated, suggesting that narrowing the focus of spatial attention to one of the two stimuli has impaired object-to-object contextual integration processes (see Figure 2, right). A statistically significant between-subject interaction was obtained when comparing the results of the two experiments. These findings imply that the allocation of spatial attention to an object is important and perhaps even critical for associating that object with a nearby item.

## 6. Is Spatial Attention Necessary for the Processing of Categorical Relations?

The findings reviewed above suggest that the processing and understanding of the spatial (contextual) relations among objects largely rely on attentional capacity. However, what about other types of relations? Note that objects sharing a common space in real life often (albeit not always) belong to the same overall category, resulting not only in semantic and spatial proximity (i.e., a common global context) but also in a relative perceptual similarity among the objects. For instance, a sofa in a living room is typically seen near a couch or a table, and a truck on the road is viewed near some other cars or vehicles. Since an attended truck shares more visual features with a car than with a traffic light or a street sign, this relative similarity, or feature overlap among vehicles, may aid the processing of the car. That is, the truck’s visual features may prime, or activate, the shape and features of the car, reducing perceptual ambiguity and allowing a better understanding of the overall scene even if the car appears in an unattended region. Is this indeed the case? Are categorical relations among nearby objects processed, and can the items prime each other, when a certain object is positioned at the outskirts of focal attention?

**Figure 3:**
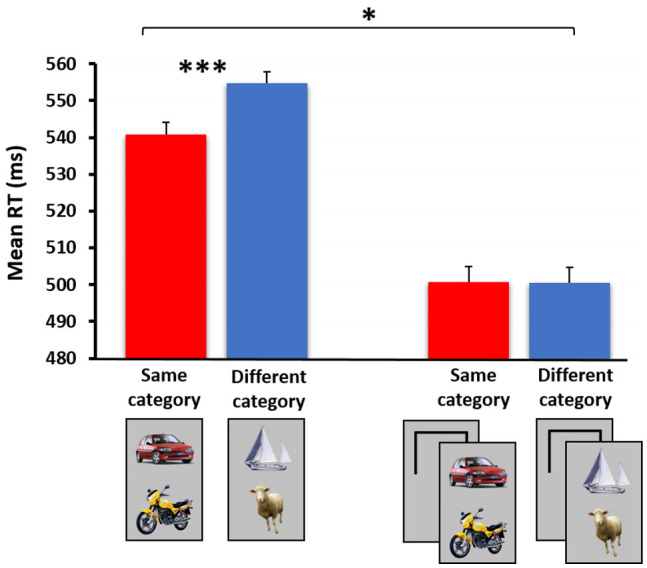
The categorical relation effect among the nontarget stimuli in the basic experimental version (i.e., both stimuli are attended, left) vs. the spatial cuing experimental version (i.e., only one of two stimuli is fully attended and relevant to task requirements, right). The black frames on the right denote the cue, briefly appearing prior to stimulus presentation in an upper (50%) or a lower location. A statistically significant between-subject interaction was obtained when comparing the results of the two experiments (N = 21 in each). * *p* < 0.05, *** *p* < 0.001. The full results are presented in [107].

In a follow-up study [107], I used a paradigm similar to the one described above, in which pairs of objects either belonged to same or to different superordinate categories (e.g., two vehicles vs. a vehicle and an animal, respectively). As in the previous study, participants were instructed to determine as fast as possible whether a nonsense shape appeared in a briefly flashed display (i.e., nonsense shapes functioned as ‘targets’, while the two real-world object categories were considered, unbeknownst to participants, as ‘nontargets’). Here, too, in the baseline study both stimuli were attended, and it was hypothesized that among the real-world object trials, same-category pairs would be processed faster and more accurately than different-category pairs. This hypothesis was based on previous priming effects shown among pairs of pictorial stimuli belonging to the same superordinate category (e.g., [92,108,109,110,111]). Indeed, when both stimuli were presented within the main focus of visual attention, a categorical relation effect was observed, in which same-category nontarget pairs were processed more efficiently than different-category ones (see Figure 3, left).

We now turned to the more interesting experimental paradigm in which spatial attention was manipulated, by using a spatial cue that summoned attention to *one* of the two stimuli prior to their appearance. As in our previous series of studies, in this ‘unattended’ experimental version participants focused on and responded to one of two images, while its counterpart image functioned as an irrelevant distractor falling outside the main focus of spatial attention. Once again, to the extent that a meaningful categorical relation effect was observed under these conditions, one could infer that the processing of stimuli’s categorical relations did not critically rely on focal attention. If, however, no categorical relation effect was observed when one of two stimuli was deprived of focal attention, it could be reasonably concluded that the extraction of visual/conceptual relations was not an attention-free process. The results clearly supported the latter option (see Figure 3, right), yielding a statistically significant interaction of categorical relation by experiment. Our findings suggested that, as in our previous study using contextually (or spatially) related object pairs, perceiving the categorical relations among objects necessitates spatial attention.

**Figure 4:**
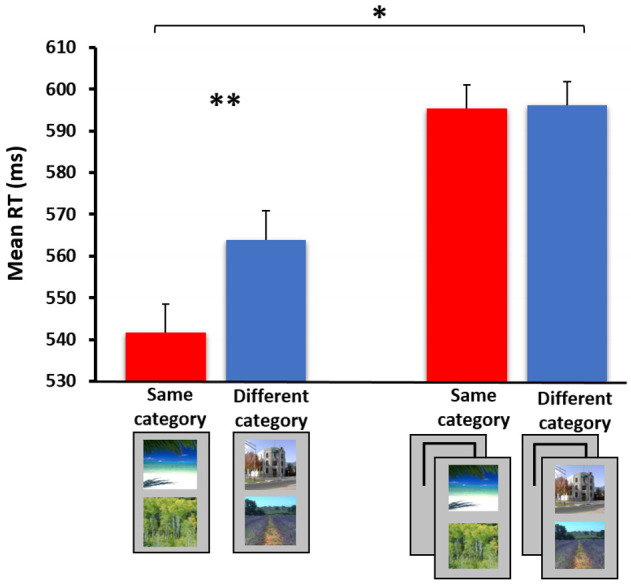
The categorical relation effect among scenes in the basic experimental version (i.e., both stimuli are attended, left) vs. the spatial cuing experimental version (i.e., only one of two stimuli is fully attended and relevant to task requirements, right). Here, two images depicting a nature scene form the same-category condition, while an image of a nature scene paired with an urban scene forms the different-category condition. A statistically significant between-subject interaction was obtained when comparing the results of the two experiments (N = 18 in each). * *p* < 0.05, ** *p* < 0.01. Note that the apparent overall increase in participants’ RTs in the spatial cuing experimental version relative to the uncued (basic) experimental version is non-significant (*p* > 0.1, see elaboration below). The full results are presented in [112].

Importantly, the processing of the categorical relations can be tested not only among individual objects, but also among larger-scale stimuli such as whole scenes. As mentioned earlier, scenes can be rather easily categorized at brief visual exposures (e.g., [23,29]). However, the extent to which the categorical relations among two simultaneously presented scenes are rapidly processed and the degree to which these rely on attentional capacity are still largely unknown. Using a similar paradigm to the one described above, we demonstrated a dissociation between processing scene categorical relations under ‘attended’ and ‘unattended’ conditions [112]. These findings suggest that, like individual objects, the understanding of scene relations is not attention-free (see findings in Figure 4). Furthermore, a similar pattern of results was observed when testing the associative relations between a central foreground object (e.g., a car) and its background scene (e.g., a street) (paper in preparation). The lack of an object–scene association observed when participants focused their attention on the object only (in contrast to focusing attention on both object and background scene) resonates the bulk of null findings found with the scene–object consistency paradigm when an item (either an object or its background) was presented at a peripheral location and was minimally attended (see review above).

One issue warrants further discussion. Note that inserting a spatial cuing manipulation is expected to reduce overall latencies due to focused spatial attention on one instead of on two stimuli. Namely, one would predict overall longer RTs in the basic experimental version (where both stimuli are attended) than in the spatial cuing experimental version (where only one of two stimuli is attended). Indeed, such a result pattern was obtained in the studies examining categorical relations among objects (see Figure 3), and among objects and their background scenes (paper in preparation). When using scene pair stimuli, however, an unexpected, non-significant trend in the opposite direction was obtained (Figure 4). We suspect that this trend may have emerged from individual differences in baseline RT performance within the different groups participating in the basic/spatial cuing experiments. Importantly, regardless of general cuing effects and/or group baseline RT, the fact that an experiment by categorical relation interaction was consistently observed across studies suggests that the dissociation between the results of the ‘attended’ and ‘unattended’ experimental versions is robust.

Taken together, the results from several series of studies using the spatial cuing paradigm suggest that the visual–semantic associations between objects and/or scenes are rapidly extracted under fully attended conditions. If, however, one or more stimuli fall ‘outside’ the main focus of spatial attention, processing these associations is largely impaired.

## 7. Feature-Based Attention: The Unique Status of Prioritized (Task-Relevant) Stimuli

The findings reviewed above referred to incidental relational processing among object and scene stimuli, namely, the processing of contextual and categorical relations among stimuli that are not actively searched for or explicitly defined as targets of interest. An example mentioned earlier is the case in which participants were asked to detect a nonsense shape, while our analysis focused on relational processing within trials containing real-world object stimuli. We focused on these ‘non-prioritized’ items since much of the debate concerning the role of spatial attention in associative processing refers to stimuli of this sort. For instance, in many of the scene–object consistency paradigms reviewed earlier, participants were asked to passively view a scene as a preview to a later memory test (e.g., [53]), or to detect an arbitrary probe stimulus appearing subsequent to scene and object exposure [55]. Similarly, paradigms assessing object-to-object relations used semantically associated objects as *irrelevant* backgrounds to target letters [100]. Relations between objects, or between an object and its background scene, were largely irrelevant and orthogonal to participants’ task requirements in these studies.

When discussing stimuli that are explicitly defined as to-be-detected targets, however, a very different picture emerges. In fact, there is a general consensus that this type of stimulus enjoys a special status both ‘inside’ *and* ‘outside’ focal attention. Thus, for instance, when engaged in a category detection task in which participants are requested to detect ‘a piece of furniture’, there are good chances that an image of a table or a chair would be spotted and recognized even when appearing at the outskirts of spatial attention. Presumably, participants tune themselves to the visual features that characterize the specific class of targets (e.g., square-ish, wooden items), and this tuning or activation of task-relevant features enables efficient detection even within underprivileged spatial locations. Indeed, the tuning process associated with current goals and/or task requirements is the hallmark of theories emphasizing *feature-based attention* [113,114,115] (see also [116,117]). Feature-based attention is the mechanism thought to select information on the basis of prioritized, non-spatial features (such as color or orientation). Presumably, it spreads across global areas of the visual field, and is therefore largely independent of spatial-attention selection mechanisms (see, e.g., [113,118,119]).

**Figure 5:**
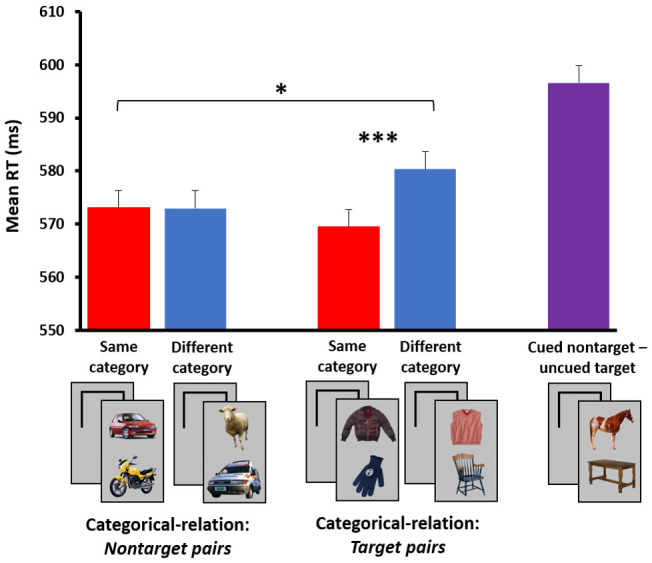
The *categorical relation effect* (red vs. blue bar) within the Target and Nontarget pair conditions when using a modified spatial cuing paradigm with four object categories. The two target categories in the present example are furniture and clothes, while the nontarget categories are animals and vehicles. The results presented here converge on previous findings in showing no categorical relation effect among the nontarget trials, when one of two stimuli appears outside the main focus of spatial attention. Interestingly, however, a significant categorical effect is now observed among the target trials, forming a clear dissociation between the two types of stimuli. A statistically significant within-subject interaction is obtained when directly comparing the results of these two pair conditions (N = 24). Notice also the *target distraction effect,* which is computed as the difference between trials containing a cued nontarget and an uncued target (rightmost purple bar) and the nontarget, different-category condition (left blue bar). Both types of trials contain objects taken from different categories, and in both trials a negative response (‘no target’) is required at the cued location. However, when comparing the RTs between these two trial conditions, significantly slower responses are obtained when the uncued (spatially irrelevant) location contains a to-be-detected target than when it comprises a nontarget. Possible explanations for this interference (or flanker-like) effect are described in the main text. * *p* < 0.05, *** *p* < 0.001. The full results are presented in [107] (Experiment 4).

Do we find any evidence for feature-based selection in our spatial cuing paradigm? As mentioned above, participants in all of our experiments were instructed to detect a target category (e.g., nonsense shapes, or in some studies a real-world category such as furniture), while the other two image categories served as nontargets (vehicles, animals). No incidental categorical relation or spatial consistency effects were seen among the nontargets when one of two stimuli was unattended. Presumably, analysis of the unattended stimulus was attenuated to a degree that did not allow a meaningful object-to-object (or object-to-scene) relational processing. However, if the uncued location contained an object belonging to the *target* category, it managed to create a robust distraction effect when paired with a nontarget object positioned in the cued (attended) location. To illustrate this distraction effect, let us assume that participants were instructed to detect a piece of furniture (i.e., the target category). On one trial, the cued location contained a nontarget (non-prioritized) animal image, while the uncued location contained a target image (i.e., piece of furniture); on another trial, both locations contained nontarget images that differed in category (e.g., an animal and a vehicle). Note that both types of trials contained objects taken from different categories, and in both trials a negative response (‘no target’) was required at the cued location. However, when comparing the RTs between these two trial conditions, significantly slower responses were obtained when the uncued (spatially irrelevant) location contained a to-be-detected target than when it comprised a nontarget stimulus (see ‘target distraction effect’ in caption of Figure 5). This ‘flanker-like’ interference effect may originate from two sources, which are not necessarily mutually exclusive. First, similar to typical flanker tasks, the appearance of the target stimulus in an irrelevant location *conflicted* with the required response to the nontarget stimulus at the relevant location. Namely, the uncued target potentially activated a response (‘target’) that was incongruent with the response (‘no target’) elicited by the cued nontarget. In contrast, both stimuli in the different-category nontarget condition activated identical (‘no target’) responses, thus the difference between these conditions reflected a strong response–conflict (or response–incongruency) effect. (Note that no response–conflict was involved in the categorical relation effect previously described, since both same- and different-category conditions comprised nontarget stimuli only). Second, due to its importance to task requirements, the target object positioned in an irrelevant location may have elicited a rapid, involuntary *shift* of attention to its location. Namely, the distraction effect may have resulted from an inadvertent attentional capture (or ‘slippage’) caused by the prioritized target, while no such capture was caused by the nontarget. It is important to mention that although several researchers have proposed attention-shift accounts for situations like this [102,120,121,122], given the extremely short exposure durations used in our studies, a more probable account is that of a parallel processing of the to-be-detected target at the uncued location [104,123,124,125] (see also [126,127,128]). Critically, regardless of the specific underlying mechanism, the strong interference elicited by the target is a clear demonstration of a feature-based selection mechanism that likely reduces the threshold of task-relevant features (or increases their activation levels), allowing prioritized stimuli to ‘break’ through the spatial attention gating mechanism and access high-level processes (e.g., [126,129,130,131]).

Yet, can our findings further inform us about object-to-object *relational* processing, when a prioritized stimulus appears at the outskirts of spatial attention? Clearly, a single target is detected even when positioned in an unattended location, but can we assess visual–semantic (e.g., categorical) processing among *pairs* of target items, as measured among the nontarget stimuli? To answer this question, we ran a modified version of the spatial cuing experiment, in which the categorical relation effect was measured within both nontarget and target pair objects ([107], Experiment 4). Rather than using one target category, we now used two target categories, resulting in overall four real-world object categories—two assigned to the nontarget conditions (e.g., animals, vehicles) and an additional two assigned to the target conditions (e.g., furniture, clothes) (target and nontarget identities were counterbalanced across participants). Instructions were, for instance: ‘if there a piece of furniture or an article of clothing in the cued location press ‘1’, otherwise press ‘2’’. Using this modified paradigm, we could now measure the categorical relation effect among the target items, while controlling for response–conflict factors, as stimuli in the same- and different-category conditions activated the same ‘target’ response (a congruent situation). Additionally, within these target pairs, the uncued location always contained a target stimulus, thus any potential ‘slippage’ of spatial attention to the task-relevant target was held constant across conditions and could not account for a categorical relation effect. The modified paradigm thus enabled a direct comparison of the categorical relation effect among target and nontarget trials.

The results showed a nice dissociation between the two types of object pairs: while no categorical relation effect was observed with the nontarget stimuli (replicating our previous results), a statistically significant effect was yet observed with the target objects (see Figure 5). A categorical relation (Same/Different) by target presence (Target/Nontarget) interaction further emphasized this dissociation, suggesting that prioritized stimuli formed a unique class of items that were processed and linked to other (attended) stimuli, even when appearing outside the main focus of visual attention.

Note that as in previous studies, an additional condition containing a cued nontarget (e.g., animal) and an uncued target (furniture) further revealed a robust ‘target distraction effect’ when compared to the different-category nontarget condition (see rightmost purple bar, compared to the left blue bar in Figure 5). As explained above, factors such as response–conflict and/or attention capture (by the uncued target) may have contributed to this large effect.

## 8. Summary and Future Directions

Accumulating research over the years has shown that visual and semantic associations within a scene can be rapidly extracted during a brief glimpse. The extent to which such relational processing requires attentional capacity, however, has been largely disputed. In a series of studies, I tried to assess the necessity of spatial attention in visual–semantic associative processing, while tightly controlling for the spatial and temporal aspects of visual attention. The findings from these studies consistently demonstrated the processing of object–object and object–scene relations, when non-prioritized (nontarget) stimuli were attended. Focusing the ‘spotlight’ of attention on a single stimulus while leaving its counterpart pair item at the outskirts of attention, however, largely eliminated this relational processing. Importantly, when examining pairs of prioritized (target) stimuli, the associative effects were observed even when an object was positioned outside the main focus of spatial attention. Our findings therefore portray an important dissociation between task-irrelevant and task-relevant items: while the former require spatial attentional resources in order to be linked to stimuli residing inside the attentional focus, the latter may affect high-level recognition and associative processes via feature-based attentional mechanisms that are largely independent of spatial attention.

Note that the research reviewed in this manuscript focused on situations in which spatial attention was confined to a specific location, within a very limited time window. As mentioned earlier, this was meant to prevent an inadvertent attention shift or ‘slippage’ of the attentional focus to an unattended location. When using long exposure durations and/or a distributed attention mode, however, a very different pattern of results may be obtained. Indeed, we believe that while the processing of task-irrelevant semantic information is largely attenuated outside focal attention, coarse processing (e.g., of a scene’s ‘gist’) may resume under distributed attention conditions (e.g., [101]). Relatedly, some recent studies have emphasized the importance of semantic meaning to attentional guidance, arguing that attention shifts are in fact mediated by the detection of meaningful areas (or objects) within a scene [32,33]. Note that these studies have not confined visual attention to a specific task-relevant location, and they have typically allowed long scanning durations of the scene stimuli. In our follow-up studies, we intend to directly contrast focused attention and distributed (i.e., spread) attention situations and their impact on high-level relational processing. In addition, we will manipulate some temporal aspects of attention by allowing, e.g., prior exposure to an irrelevant stimulus. We believe that when allowing spatial attention to spread over a large region in the visual field, and/or when enabling serial shifts of the attentional focus (e.g., during visual search), relational processing will take place even among stimuli that are strictly task-irrelevant. Using less stringent settings in which attention and the eyes can freely scan a visual display may thus reveal different findings than the ones observed in the present series of studies. Future research will carefully manipulate these temporal and spatial aspects in order to better understand high-level associative processing during natural scene viewing.

## Data Availability

No new data were created or analyzed in this study. Data sharing is not applicable to this article.
